# Occipital bone defect caused by neurofibromatosis type I: A case report

**DOI:** 10.1097/MD.0000000000034413

**Published:** 2023-07-21

**Authors:** Yiquan Wang, Shuyue Song, Zhen Li, Yujing Zhao, Junjie Miao, Zhe Wang

**Affiliations:** a School of Clinical Medicine, Weifang Medical University, Weifang, China; b Department of Neurosurgery, Weifang People’s Hospital, Weifang, China.

**Keywords:** neurofibromatosis I, skull bone defect, surgery

## Abstract

The synergistic effect between nonmalignant lesions can also cause a serious impact on patient survival. This disease involves different organ systems and presents with a variety of clinical manifestations, such as schwannoma, depigmentation, low-grade glioma, and skeletal abnormalities. We report a case of type I neurofibromatosis with an occipital bone defect.

**Patient concerns:**

We report a case of a 50-year-old man with type I neurofibromatosis with occipital bone defect.

**Diagnosis:**

The patient was accepted by the local hospital due to sudden right upper limb weakness accompanied by jitter without recognizable cause or inducement. A computerized tomography scan at a local hospital suggested subcutaneous neurofibromatosis with a left occipital cranial defect with thinning bone. On admission physical examination, diffuse multiple masses could be seen throughout the body and head of different sizes and composed of soft and tough textures. The largest one was located in the posterior occipital bone, approximately 8*8 cm in size, with a child tumor and tough texture. Multiple café-au-lait spots could be found on the chest and back, and multiple freckles can be seen in the armpit. The patient underwent surgery. Postoperative pathology showed a spindle cell tumor, which was determined to be neurofibromatosis type I according to immunopathology and clinical data.

**Interventions:**

The patient was admitted for surgical treatment. During the operation, the scalp mass was completely abducted and the tumor tissue at the skull defect was sharply separated. Postoperative pathology showed that the peripheral margin and the bottom margin were cleaned.

**Outcomes:**

Computerized tomography and magnetic resonance imaging showed that the tumor was completely. There were not any surgical complications. The patient recovered well, was cured and was dismissed from the hospital.

**Lessons:**

The synergistic effect between nonmalignant lesions can also cause a serious impact on patient survival to encourage early medical intervention. The clinical presentation of neurofibromatosis type I am usually nonmalignant, and in this case, involvement of the skull with bone defect is very rare. Therefore, it is necessary to accumulate relevant cases, reveal the pathogenesis of the disease, predict the development and outcome, and provide more evidence for early therapeutic intervention of similar patients in the future.

## 1. Introduction

Neurofibromatosis type I is a complex autosomal dominant disorder caused by germline mutations in the NF1 tumor suppressor gene.^[1]^ The incidence varies substantial between countries, with a global average of approximately 1 in 3000.^[2]^ The clinical manifestations, types of complications, and speed and degree of progression of the disease vary from person to person and gradually develop throughout life. At present, due to the lack of effective therapeutic methods, clinical management typically involves monitoring and symptomatic treatment.^[1]^

Deletion of the key NF1 gene in neurofibromatosis type I in different cell types leads to different manifestations of cell dysfunction.^[1]^ This disease involves different organ systems and presents with a variety of clinical manifestations, such as schwannoma, depigmentation, low-grade glioma, and skeletal abnormalities. Schwannomas are benign tumors closely related to the spinal nerves, peripheral nerves and cranial nerves that compress and push surrounding structures but are rarely invasive and destructive. Skeletal abnormalities include scoliosis and tibial dysplasia^[3]^ and rarely lead to bone defects. We report a case of type I neurofibromatosis with an occipital bone defect.

## 2. Case presentation

**Figure 1.**
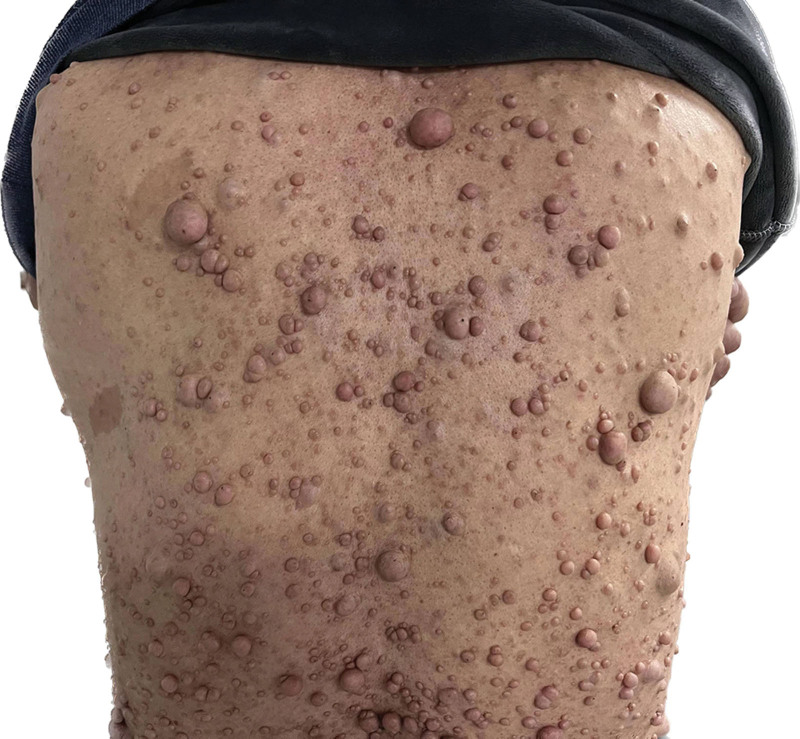
Multiple neurofibromas and milky coffee spots on the back.

**Figure 2.**
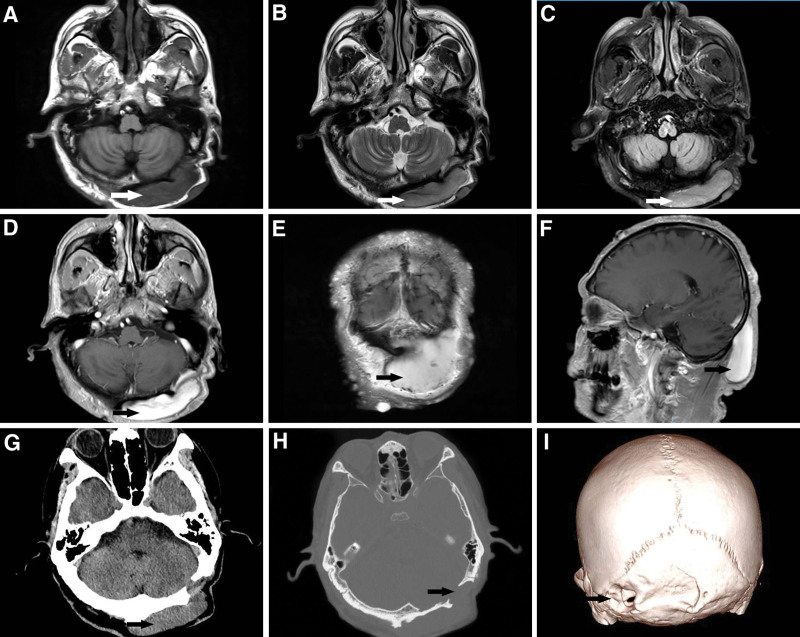
Preoperative MRI scanning for neurofibromatosis type I: T1-weighted, T2-weighted, diffusion-weighted imaging, T1-weighted enhanced axial, coronal, sagittal (A–F); CT plain scan, CT bone window, 3D reconstruction (G–I). CT = computerized tomography, MRI = magnetic resonance imaging.

**Figure 3.**
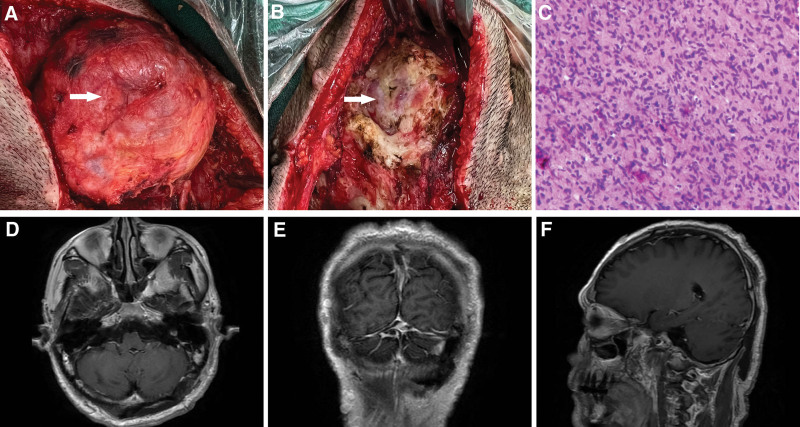
Interpretative findings of great occipital schwannoma: great occipital schwannoma, occipital bone defect (A and B); Postoperative pathology: HE staining showed fusiform cell tumor (C); Postoperative T1-weighted enhancement: axial, coronal and sagittal views showed complete tumor resection (D–F).

The patient was a 50-year-old male. Approximately 40 years ago, there were multiple masses all over the body without obvious reasons and inducement, mainly in the occipital region, that gradually increased in number but never received great attention. There was no obvious headache, nausea and vomiting, or body convulsions. Three days prior, the patient developed right upper limb weakness accompanied by jitter without recognizable cause or inducement. A computerized tomography scan at a local hospital suggested subcutaneous neurofibromatosis with a left occipital cranial defect with thinning bone. The patient had a history of hypertension for 4 years, and the highest systolic blood pressure was approximately 140 mm Hg. Valsartan and atenolol were administered orally as usual, and his blood pressure was controlled effectively. He acknowledges the hospital on January 12, 2023. On admission physical examination, diffuse multiple masses could be seen throughout the body and head of different sizes and composed of soft and tough textures (Fig. 1). The largest one was located in the posterior occipital bone, approximately 8*8 cm in size, with a child tumor and tough texture. Multiple café-au-lait spots could be found on the chest and back (Fig. 1), and multiple freckles can be seen in the armpit. The patient has completed head computerized tomography (Fig. 2G–I) and head magnetic resonance imaging (Fig. 2A–F) examinations. The patient underwent surgery. Intraoperatively, the mass was deemed to be dermal with a soft capsule, rich in blood supply and gray-red in color (Fig. 3A). The mass was regarded as closely related to the abnormally swollen occipital greater nerve, so it was removed. The capsule completely encircled the mass, and the tumor was completely resected, ultimately measuring approximately 8*8*3 cm. During the operation, erosion and thinning of the left occipital bone was noted, and part of the occipital head tumor had eroded the posterior fossa dura, forming a skull defect of approximately 2.5*2.5 cm in size (Fig. 3B). The tumor tissue at the skull defect was dissected with sharp separation, removing a mass measuring approximately 2.0*0.5 cm while keeping the dura intact and preventing cerebrospinal fluid leakage. Postoperative pathology showed a spindle cell tumor (Fig. 3C), which was determined to be neurofibromatosis type I according to immunopathology and clinical data. The patient postoperative magnetic resonance imaging scan of the head showed complete resection of the tumor (Fig. 3D–F). The patient recovered well, was cured and was dismissed from the hospital.

## 3. Discussion

The gene responsible for neurofibromatosis type I is NF1, whose mutation or deletion leads to a decrease in neurofibromin, leading to cellular dysfunction. Neurofibromin is expressed in a variety of cell types and plays an important role in inhibiting cell proliferation, similar to the protein family of negative regulators of the RAS proto-oncogene.^[4,5]^ Reduced expression of neurofibromin leads to increased cell growth and survival through overexpression of RAS,^[4,5]^ manifesting as pigmented lesions, tumors, and abnormal bone formation. In addition, the cognitive level of the affected population plays an important role in the progression and outcome of the disease.

The clinical manifestations of neurofibromatosis type I vary in terms of pathogenesis. Some are caused by NF1 haploid deficiency, while others require basal cell inactivation or other gene changes. For example, double-allele NF1 inactivation is required for the development of CALMs and neurofibromin, and P53 mutation is also necessary for the formation of malignant peripheral nerve sheath tumors.^[1]^ In this case, postoperative pathology revealed a spindle cell tumor (nonmalignant), so malignant invasion of the skull can be ruled out.

There are 3 hypotheses regarding the mechanism of skull defect formation in patients with NF1.

The deletion of the NF1 gene in both osteoblasts and osteoclasts can lead to functional disorders in both kinds of cell, thus leading to disorders of bone formation and destruction: Abnormal bone mineralization has been demonstrated in osteoblasts from NF1-deficient mice, resulting in decreased bone mineral density and increased fracture risk^[6]^; In NF1-deficient osteoblasts, certain irreducible proteins that induce mesenchymal stem cells to differentiate into osteoblasts are significantly reduced, thus affecting the process of bone remodeling^[6]^; The decrease in neurofibromin in NF1-deficient osteoblasts leads to changes in intracellular substance synthesis and enzyme activity through the influence of specific transcription factors, ultimately interrupting bone maintenance^[7]^; Nf1-deficient osteoblasts can promote the transformation of osteoclasts into active osteoclasts through certain cytokines and eventually lead to osteoclast disorders.^[1]^

In stress osteolysis, skull defects are related to pressure transmitted by surrounding tissues. Osteolysis can occur when bone cells are subjected to peripheral stress, which is similar to RAS pathway activation. Examples include orbital plexus neurofibroma or peripheral dural dilatation.^[8]^

In nonstress osteolysis, bone malformations are caused by defective mesoderm and neuroectoderm development. It is more common in the orbital part of the sphenoid and less common in the occipital region and ipsilateral mastoid air chamber hypoplasia and hypoinflation.^[9]^

In this case, it was not known whether the patient had congenital bone loss, and the possibility of a malignant tumor was excluded. It was speculated that the bone defect in the patient might be caused by a variety of nonmalignant factors, among which the most likely was a combination of bone damage and stress osteolysis. The patient may have had long-term dysfunction of bone formation and osteopenia, while the diseased occipital bone was subjected to the long-term pressure effect of the great occipital nerve sheath tumor, resulting in the occipital bone defect.

In this case, the patient original intention in seeking medical treatment was not related to the local and systemic tumor symptoms. The limitations of the patient cognition, culture, willingness to seek medical treatment and other factors provided a foundation for the progression of the tumor. Therefore, under the synergistic effect of various nonmalignant lesions, an occipital bone defect was eventually caused, and the disease could have progressed further if left unchecked.

At this stage, the tumor had formed a potential threat to the patient life safety, and if it had continued to develop intracranially, it would have had a profound impact on the patient quality of life and even survival. Compared with healthy people, head trauma is more dangerous to such patients and even life threatening. When trauma occurs, if the force is located around the bone defect, the protection and buffering effect of the occipital bone on the intracranial tissue is lowered due to the absence and weakness of the occipital bone here. Here, the tumor can directly act on the intracranial space due to the application of external forces, resulting in an acute increase in intracranial pressure and cerebral hernia. Additionally, the patient weak occipital bone would be more likely to fracture and shatter during the trauma, and the fractured plate could damage brain tissue and blood vessels and cause acute intracranial hemorrhage.

The purpose of this case report is to describe this special clinical manifestation, explore and predict the development and outcome of the disease to reveal its pathogenesis in the future, and demonstrate that the synergistic effect between nonmalignant lesions can also cause a serious impact on patient survival to encourage early medical intervention.

## Author contributions

**Conceptualization:** Yujing Zhao, Zhe Wang.

**Data curation:** Yiquan Wang, Shuyue Song, Zhen Li, Junjie Miao.

**Project administration:** Zhe Wang.

**Software:** Yiquan Wang, Shuyue Song, Zhen Li, Junjie Miao.

**Writing – original draft:** Yiquan Wang, Shuyue Song, Junjie Miao.

**Writing – review & editing:** Zhen Li, Yujing Zhao.
